# Supplementary material for: Rapid turnover of life-cycle-related genes in the brown algae
Source: Genome Biol. 2019 Feb 14;20:35. doi: 10.1186/s13059-019-1630-6 (PMC6374913; doi:10.1186/s13059-019-1630-6)
Supplement: Supplementary file 3 — Protein alignments, corresponding Gblocks html file and CDS sequences for the lineage conserved gametophyte-biased genes. (ZIP 2109 kb) [file 13059_2019_1630_MOESM3_ESM.zip › Con_GA_alignments/Ec-05_005720.aa_ali.fasta-gb.htm]

Ec-05\_005720.aa.fasta.trans.aa\_ali.fasta


## Gblocks 0.91b Results

Processed file: **Ec-05\_005720.aa.fasta.trans.aa\_ali.fasta**  
Number of sequences: **2**  
Alignment assumed to be: **Protein**  
New number of positions: **272** (selected positions are underlined in blue)

```
                         10        20        30        40        50        60
                 =========+=========+=========+=========+=========+=========+
Ec-05_005720     MDTPATNRPSGALEPELSPMSKKVRHFEKYSRKGGRWSWKRPSYERDVVNLVKTERRIEE
MSTRG.29527.1    ------------------------------------------------------------
                                                                             


                         70        80        90       100       110       120
                 =========+=========+=========+=========+=========+=========+
Ec-05_005720     GIASALTEVDAVEAGLRYFDELIIPQNKHVGIQQQLISSNGQDESNGVDTRKASRDGPGS
MSTRG.29527.1    ------------------------------------------------------------
                                                                             


                        130       140       150       160       170       180
                 =========+=========+=========+=========+=========+=========+
Ec-05_005720     RSKGPRAQGGRSRDEGRAGFTRSEGAKLRDYVAAKKNVVGVGNSIMTSSDRAVSECVDLK
MSTRG.29527.1    ------------------------------------------------------------
                                                                             


                        190       200       210       220       230       240
                 =========+=========+=========+=========+=========+=========+
Ec-05_005720     LTLKQRLDNYDRQRGINVTALKDEEKSEAQLVSFKQQNNHEMKAKHSKRSGGGTIGSNVE
MSTRG.29527.1    ------------------------------------------------------------
                                                                             


                        250       260       270       280       290       300
                 =========+=========+=========+=========+=========+=========+
Ec-05_005720     GGNQGGMIAPCCEGGATDAGSVGGILARSLGNAGTRRRRRMTEGGGKGRSIPGSNQDKEV
MSTRG.29527.1    ----------------------------------------------GDNSIIGSSQQNGG
                                                                  ###########


                        310       320       330       340       350       360
                 =========+=========+=========+=========+=========+=========+
Ec-05_005720     SAPQSRWRSWNEPPPRALLTAEEIRSASAAKTTDERRRQPPPPE-VIAEIIPPFRSRRRF
MSTRG.29527.1    SATRSRWKSWNEAPPPALLGAGGVRNLSATQNGDGRHPQHMSATGASSEVLPPASSRRRA
                 #######################################         ########### 


                        370       380       390       400       410       420
                 =========+=========+=========+=========+=========+=========+
Ec-05_005720     R-TPSGNSNASDEKGKPTEHAAAGVSLESGEGEKRRDGCNGE-------GLVSTTTAAPS
MSTRG.29527.1    GGVNSISASTSSTSALTVKSSSAGETSATSVSSAKRLSVGGDKRMGASAGEVPPSAASSS
                                                                  ###########


                        430       440       450       460       470       480
                 =========+=========+=========+=========+=========+=========+
Ec-05_005720     SSRR-TAAGGVPLVPRVATLATPPATEKNKTEATKKGKDFDPDQLECVRRHEASIAEKER
MSTRG.29527.1    SSRRSSAASGVPLVPRVAMLAAACANRSEEAEAARKGKEFDPDQLECVRRHVTSMAERER
                 ####  ######################################################


                        490       500       510       520       530       540
                 =========+=========+=========+=========+=========+=========+
Ec-05_005720     MVHSIREEAEKRTFFRARPLPAFLDGDGAVPWSVG--DLTSKGCGDARGKGGKSGRGSAA
MSTRG.29527.1    RLRSMRSEAEKKTLFRARPLPAFLDGGGGVATDSDRRFLSSRGGSGARRKRDGAGQGQAT
                 ##############################        ######################


                        550       560       570       580       590       600
                 =========+=========+=========+=========+=========+=========+
Ec-05_005720     GTGLGEAGTRPELLAALEQDEEAQALVEKIRKINPRADIASIVLQSTPRTPLSLPLTSRI
MSTRG.29527.1    SVGSGLAGSSPELLEALEQDEEARALVEKIRKINPRVDIASIVRQSTPRTPLPLQPTTRT
                 ############################################################


                        610       620       630       640       650       660
                 =========+=========+=========+=========+=========+=========+
Ec-05_005720     NNGGGRVTRPFFSQGGVLTPAGNATHTATTAATGLSWSKNNTASRPLMSVGVSDQSTPGL
MSTRG.29527.1    SDGDCGVT----------------------------------------------------
                 ########                                                    


                        670       680       690       700       710       720
                 =========+=========+=========+=========+=========+=========+
Ec-05_005720     KSEHKSPQSVVRVDGSDARERSEGRAEESHVTRVSGICERKPEDGQERGMLGTGDQQVRH
MSTRG.29527.1    ------------------------------------------------------------
                                                                             


                        730       740       750       760       770       780
                 =========+=========+=========+=========+=========+=========+
Ec-05_005720     HMATGASASATSGPASAPENDGEDSGDPRCVTESRRRSLMGRMLESGMLERQKEWAKARS
MSTRG.29527.1    ------------------------------------------------------------
                                                                             


                        790       800       810       820       830       840
                 =========+=========+=========+=========+=========+=========+
Ec-05_005720     KKVLESRRQVEAAKEQEGKGDALVAGLSGRSWSKAKTEHLRSLERQRKEEETREEIREAR
MSTRG.29527.1    ------------------------------------------------------------
                                                                             


                        850       860       870       880       890       900
                 =========+=========+=========+=========+=========+=========+
Ec-05_005720     RRASELRAERERESAERRATEIADTLAAAADAVGAGTGRESRTAPNSRGGGGRRRLRRPP
MSTRG.29527.1    ----------------------------------------------------------PR
                                                                           ##


                        910       920       930       940       950       960
                 =========+=========+=========+=========+=========+=========+
Ec-05_005720     PPRRARSHAGDEGHVNRTGDGGGIVSMTANERRQKTEGETSPDGGNHVTPVVATEAKNDL
MSTRG.29527.1    PSSRGATVAGTS------------------------------------TVLASASS----
                 ##########                                                  


                        970       980       990      1000      1010      1020
                 =========+=========+=========+=========+=========+=========+
Ec-05_005720     SEDSFYRVAVEKIMEKERLGVASHGLLGGSGSGGGGGDGGDGGKSGAGSSGRRRTSCSGR
MSTRG.29527.1    ------------------------------------------------------------
                                                                             


                       1030      1040      1050      1060      1070      1080
                 =========+=========+=========+=========+=========+=========+
Ec-05_005720     SHDRSRKGAAAGPLLFHLPRGAAAGRRTRTKSGAPMHQSQARGANGTPNSTVGRRRSWEI
MSTRG.29527.1    --------------------------------------ARSMCADALPSVSVGTT-AGGL
                                                            ##########       


                       1090      1100      1110      1120      1130      1140
                 =========+=========+=========+=========+=========+=========+
Ec-05_005720     QTSRTSERHAVTPPTLSADRALARTSSLLSLYEAQSVEHVAGIRRDNSGPYLDDDDALGP
MSTRG.29527.1    QSTDVPGKQQSLPPSMDPD-----------------------------------------
                                                                             


                       1150      1160      1170      1180      1190      1200
                 =========+=========+=========+=========+=========+=========+
Ec-05_005720     SRDHTSAGWPGGEAQDRLTLAGQEQLSVLVATSGAEKFTPSGGAAGAMDYPWSDERRASG
MSTRG.29527.1    ----------------RAT-----------------------------------------
                                                                             


                       1210      1220      1230      1240      1250      1260
                 =========+=========+=========+=========+=========+=========+
Ec-05_005720     SRGSGGGARHIVEGGTRLDAAAATVSGSPRWNDVLNIGQQLDQARGTSANEGEEHPRRRR
MSTRG.29527.1    ------------------------------------------------------------
                                                                             


                       1270      1280      1290      1300      1310      1320
                 =========+=========+=========+=========+=========+=========+
Ec-05_005720     GGDVGGGAVTRLGGRFDLWNESDDEVSVTEADRHEHHTNLERRNEHHASGSNSQACEGAG
MSTRG.29527.1    ------------------------------------------------------------
                                                                             


                       1330      1340      1350      1360      1370      1380
                 =========+=========+=========+=========+=========+=========+
Ec-05_005720     ALDSGDDGGHAMDSFLMPAQGTGFPVTAQAGGFFDAATSSADKGRLKVRDPRLFDPCSMR
MSTRG.29527.1    ------------------------------------------------------------
                                                                             


                       1390      1400      1410      1420      1430      1440
                 =========+=========+=========+=========+=========+=========+
Ec-05_005720     RRPDSDFRGTSAKPKVVDNEATRVPHFPEGRAASTGVTLLLGRRVDDEGRSFGGIGGEGR
MSTRG.29527.1    ------------------------------------------------------------
                                                                             


                       1450      1460      1470      1480
                 =========+=========+=========+=========+=
Ec-05_005720     QVHGAEEVITVMFDRSKFNEQEAHRWWLANGARIVNISDRG
MSTRG.29527.1    -----------------------------------------
```

```
Parameters used
Minimum Number Of Sequences For A Conserved Position: 2
Minimum Number Of Sequences For A Flanking Position: 2
Maximum Number Of Contiguous Nonconserved Positions: 8
Minimum Length Of A Block: 10
Allowed Gap Positions: None
Use Similarity Matrices: Yes
```

```
Flank positions of the 7 selected block(s)
Flanks: [290  339]  [349  359]  [410  424]  [427  510]  [519  608]  [899  910]  [1064  1073]  

New number of positions in Ec-05_005720.aa.fasta.trans.aa_ali.fasta-gb:  272  (18% of the original 1481 positions)
```
